# Pomegranate morpho-chemodiversity: computational investigations based on *in-vivo* and *in-vitro* screening

**DOI:** 10.1016/j.heliyon.2022.e09345

**Published:** 2022-04-27

**Authors:** Assia Ejjilani, Karim Houmanat, Hafida Hanine, Lahcen Hssaini, Kaoutar Elfazazi, Francisca Hernandez, Ilham Hmid, Rachid Razouk

**Affiliations:** a National Institute of Agricultural Research (INRA), Morocco; b Faculty of Sciences and Techniques, Laboratory of Bioprocess and Bio-Interfaces, University of Sutan Moulay Slimane, Beni-Mellal, Morocco; c Dpto. Producción Vegetal y Microbiología, Grupo de Investigación de Producción Vegetal y Tecnología, cuela Politécnica Superior de Orihuela (Universidad Miguel Hernández de Elche), Ctra. de Beniel, km 3,2, E- 03312 Orihuela, Alicante, Spain

**Keywords:** *Punica granatum* L., Phenotypic diversity, Fruit quality traits, Lipo-biochemical attributes, Juice proprieties

## Abstract

Pomegranate tree is cultivated since ancient times in Morocco, where a high genetic diversity is hosted mainly in traditional agroecosystems. Over the past decade, it regained importance through extension of cultivated area, but remains thus far little valued. To date, its genetic variability and chemodiversity have gone unheeded for many reasons, some of which are related to previous agricultural strategies. In this context, the present study aimed to screen an ex-situ collection of seven local cultivars and seven exotic varieties with regard to 50 fruit morphometric and biochemical descriptors. The results showed statistically significant variability within accessions (p < 0.01), based on all aforementioned traits, except for seed weight, with coefficients of variation greater than 49%. This indicated a high level of phenotypic diversity among the studied genetic pool. The 3D scatter plot built based on the principal component analysis displayed an interesting discrimination with regard to the genotypes' geographic origins with a total variance of about 50%. According to morphometric based-heatmap, four main clusters were identified distinguishing the typicality of some local cultivars compared to exotic varieties, mainly ‘Sefri’, ‘Bzou’, ‘Chioukhi’ and ‘Djeibi’. Traits having the highest impact on discrimination between accessions were, by order of importance, fruit weight and its dimensions, juice yield, aril yield, single aril diameter, soluble sugars (glucose and fructose) along with contents in some organic acids, including citric, palmitic, linoleic and malic acids. Potential statistically significant correlations were spotted through bi-dimensional heatmap analysis, particularly between the fruit size, shape and peel traits along with some biochemical attributes. As many areas of the species chemodiversity and functional properties are still needed to be investigated further, the results of the present study are of great interest for the species valorization and for breeding programs.

## 1 Introduction

The development of databases on agronomic and biochemical potential of pomegranate accessions is of great necessity for genetic breeding programs and selection of cultivars for industrial purpose. The available studies have mainly focused on the variability within local cultivars for the physico-chemical descriptors (Martínez et al., 2012; Fernandes et al., 2017; Hmid et al., 2018). However, few works have included other pathways of assessment such as biochemical traits that may contribute to the total variance, and lead to better discrimination (Hmid et al., 2017). The combination of both biochemical and morphometric traits can enhance the relevance of discrimination and classification of local germplasm, and making it possible to better orient its industrial valuation. Such traits could be the basis for further use and also to the improvement of industrial processes using pomegranate as raw material. All of the above aspects are guiding pillars for the enhancement and development of the species and contribute to its competitiveness globally. The answer to aforementioned issues will result in more direct future breeding programs to better meet demand of the industrial and thus the market. Many studies have largely contributed to the selection of a best set of cultivars with fairly important technological properties (Martínez et al., 2012). However, for a given cultivar and under well-defined conditions, the physicochemical composition of the fruit varies according to the edaphoclimatic conditions and the agricultural practices, which evidently influence the characteristics of the fruit (Melgarejo et al., 2000). Usually, the edible parts of pomegranate fruit accounted for 80% juice and 20% seeds. Fresh juice is composed of water (85%), sugars (10%) and a minor amount of pectin (1.5%) (Fernandes et al., 2017). Pomegranate juice is of great commercial potential as it is a source of many valuable bioactive compounds, such as phenolic compounds, organic acids, tannins (hydrolysable and condensed) and anthocyanins (Gil et al., 2000; Viuda-Martos et al., 2011; Mena et al., 2011; Akpinar-Bayizit et al., 2012; Hmid et al., 2017). Pomegranate juice as reported in previous studies can be used in soft drink formulations, other juices, syrups and fermented products (Maestre et al., 2000; Park et al., 2014).

Several studies have focused on physico-chemical characterization and phenolic composition of pomegranate juice within the local populations in different countries, such as Turkey (Gözlekçi et al., 2011; Ozgen et al., 2008), Croatia (Gadže et al., 2012; Radunić et al., 2012), Iran (Tehranifar et al., 2010; Zarei et al., 2010), Italy (Ferrara et al., 2014), Pakistan (Nafees et al., 2017) and Morocco (Hmid, 2013). These studies have shown that there is a large diversity within pomegranate species referring to the fruit shape and color alongside juice content, but also in biochemical composition of various parts of the fruit, especially in juice proprieties, regarding particularly sugars, fatty acids, amino acids and phenolic compounds (Melgarejo et al., 2000). Pomegranate is among the highly diversified and widespread horticultural fruits, known for its great nutritional benefits owing to its richness of phenolic compounds, organic acids, sugars and minerals (Malik et al., 2005; Khan and Mukhtar, 2007; Hmid et al., 2017).

Collecting, preserving and characterizing local pomegranate cultivars are valid goals, especially that some local populations are subjected to genetic erosion, mainly due to the extensive selection by farmers, using narrow criteria of selection focused mainly on fruit size and color. Such works are of great interest in regions which constitute diversification centers of the species, such as the Mediterranean basin, including Morocco (Ajal et al., 2015). In fact, the Moroccan pomegranate orchards with a total area of around 14 000 Ha, are currently based on a limited range of local cultivars and few exotic varieties (MAPMDREF, 2020). This situation makes the pomegranate sector less diversified and offering few valuation options. Thus, to obtain novel pomegranate cultivars with higher yield and better quality, it is essential to screen available pomegranate genetic resources and conserve them for better orienting breeding programs. The present study aimed at assessing an ex-situ collection of fourteen local and exotic pomegranate cultivars, on the basis of morphometric, biochemical and lipo-biochemical descriptors. To the best of our knowledge, it is an unprecedent study combining a large set of lipo-biochemical descriptors in the evaluation of pomegranate genotypes growing in Morocco.

## 2 Materials and methods

### 2.1 Plant material and experimental conditions

This work was carried out on fourteen pomegranate genotypes including seven local cultivars and seven exotic varieties, gathered in an ex-situ collection at the experimental station of the National Institute of Agricultural Research (33°56′E, 5°13′N; 499 m asl), northern Morocco (Table 1). The cultivars have a similar phenological behavior, with identical blooming and ripeness periods. They were fifteen years old and were planted following a completely random arrangement with three replicates per genotype, and a spacing of 5 × 3 m. The climate in the experimental site is of Mediterranean type, with rainy winter and dry summer. Rainfall is irregular throughout the year, with an annual average of about 470 mm, mostly occurring from October to February. The average yearly temperature is 17.1 °C. August is the hottest month with an average temperature of 36 °C, whereas January is the coldest one (5 °C). The soil was sandy clayey (43%, 10.2% and 46.8% of clay, silt and sand respectively), moderately calcareous with an average of 3.04% of CaCO_3_ and rich in organic matter with an average of 2.51%. The trees were conducted in the vase shape and irrigated daily using drip system from April to October, according to reference evapotranspiration values obtained from the weather station of Ain Taoujdate, at less than 200 m of distance from the studied collection. They received the same fertilization, namely 150, 60 and 150 kg ha^−1^ year^−1^ of N, P_2_O_5_, and K_2_O respectively, determined based on soil analysis during dormancy period (End-November). Pest control was according to local commercial practice, including copper based pesticide application in winter and fight against aphids.

**Table 1:** Denominations and geographic origins of pomegranate cultivars included in the study.

Local cultivars	Exotic cultivars	
	Cultivar	Origin
Bzou	Dwarf Semi Evergreen	USA
Chelfi	Gordo de Jativa	Spain
Chioukhi	Mollar Osin Hueso	China
Djeibi	Negro Monstrioso	Spain
Grenade Jaune	Ruby	USA
Sefri	Wonderful	USA
Sefri2	Zheri Precoce	Tunisia

### 2.2 Fruit sampling and physical traits determination

In early October, fully ripe fruits were randomly collected from each cultivar, containing 20 fruit each (5 fruits from each side of the tree canopy), while avoiding sunburned and cracked fruits. The ripeness degree was detected visually when fruits lost all traces of green color and became tinged with a yellowish or reddish color, depending on the cultivar. After harvest, the weight per fruit was recorded using a precision balance, while their dimensions (length and diameter) were determined using a digital caliper. Afterward, manually extracted arils were assessed for their dimensions and juice content, while also measuring biometric traits of seeds (weight, length and diameter) and bark (weight and thickness).

### 2.3 Juice characterization

#### 2.3.1 Chemical traits and dry matter

Titratable acidity (TA) was measured following the method described by Fernandes et al. (2017). Hence, pomegranate juice (1 mL) was mixed with 5 ml distilled water and titrated to pH 8.2 with 0.1 N NaOH. TA was calculated in grams of citric acid equivalent per liter of juice (g CAE L^−1^), using the formula: [TA = V_NaOH_.C.M.1000/V_juice_] where, V_NaOH_ is the consumption of titrant at the end point, C refers to the concentration of NaOH, M is the molecular weight of citric acid (M = 192.124 g mol^−1^) and V_juice_ is the volume of juice. The pH measurement was performed using a pH meter (Thermo Orien 3 Star). Total soluble solids content (TSS) was measured using a digital refractometer (Metteler-Toledo Gmbh. 30PX. Switzerland), calibrated by distilled water. Juice maturity index was calculated as the ratio ‘TSS/TA’. Juice dry matter was determined through freeze-drying of previously weighed juice, at -50 °C under a pressure of 0.10 mBar for 72 h.

#### 2.3.2 Juice biochemical traits

##### 2.3.2.1 Total phenolics content (TPC)

Total phenolics content was measured using the Folin Ciocalteu method described by Singleton et al. (1965). TPC extraction was performed by mixing pomegranate juice (5 mL) with 60% methanol (1:100v/v). Afterward, 300 μl of the extract solution was mixed with the Folin Ciocalteu reagent (1.5 mL) diluted 1:10 and 1.2 mL of 7.5% sodium carbonate. The mixture was left for 90 min in the dark at room temperature, before absorbance determination at 760 nm by a Safas UV-visible spectrophotometer, using gallic acid as a standard. Values were calculated as mg of gallic acid equivalent per liter of juice (g GAE L^−1^).

##### 2.3.2.2 Total flavonoids

Total flavonoids were measured by a colorimetric assay, developed by Lamaison et al. (1990). Briefly, the extract solution of juice (1 mL) was mixed with 1 mL of aluminum chloride (2%). The obtained mixture was then left at room temperature for 15 min, before absorbance measurement at 430 nm (UV-Visible Safas spectrophotometer). Values were calculated as g quercetin equivalent per litter of juice (mg QE L^−1^).

##### 2.3.2.3 Total anthocyanins

Total anthocyanin content was analyzed by the differential pH method using two buffers: potassium chloride buffer at pH 1.0 (25 mM) and sodium acetate buffer at pH 4.5 (0.4 M) (Lako et al., 2007). 0.4 mL of the extract solution of juice (1:100) was mixed separately with 3.6 mL of each of the two buffers. Mixtures were measured at 510 nm (ABS_510_) and 700 nm (ABS_700_) and results were calculated as mg of cyanidin-3-glucoside equivalent per liter of juice (mg CGE L^−1^ of juice), calculated according to the Eq. (1) below:

(1) Total anthocyanins content = (A ∗ MW ∗ FD ∗ 1000 / CAM)

Where, A: resultant absorbance, calculated by the formula: A = (ABS_510_-ABS_700_) pH_1.0_ – (ABS_510_-ABS_700_) pH_4.5_; MW: molecular weight (449.2); FD: dilution factor (10); CAM: molar absorption coefficient of cyanidin-3-glucoside (26,900).

##### 2.3.2.4 Total hydrolysable tannins

Hydrolysable tannins content was measured following the method described by Mueller-Harvey (2001). Hence, 1 mL of previously prepared juice extract was mixed in 5 mL of 2.5% KIO_3_ by stirring for 10 s. The mixture absorbance was read at 550 nm using a spectrophotometer (Safas UV-visible). Concentration in hydrolysable tannins was estimated by reference to the calibration curve using tannic acid (500–2000 mg L^−1^) as standard. The results were expressed as mg of tannic acid equivalent per liter of juice (mg TAE L^−1^).

##### 2.3.2.5 Total condensed tannins (TCT)

TCT were evaluated based on Folin Denis method as reported by Joslyn (1970). In a 100 mL flask, distilled water (75 mL) was mixed with 1 mL juice previously diluted at 1/20 ratio. Then, 5 mL of the Folin Denis reagent along with 10 mL of the saturated solution of were added to the mixture. CO_3_Na saturated solution was prepared by dissolving 43.75 g of Na_2_CO_3_ in hot water (100 mL, 80 °C). After chilling, the mixture was adjusted to 125 mL after being carefully filtrated. After mechanical stirring, the preparation was left for 30 min before optical density measurement at 760 nm. Concentrations of condensed tannins are estimated by reference to the calibration curve using tannic acid (0–0.1 g/L) as standard. Values were reported as mg of tannic acid per liter of juice.

##### 2.3.2.6 Sugar profiles

Sugars were extracted as being previously described by Hernández et al. (2015) using a Hewlett-Packard HPLC (Series 1100, Wilmington, DE, USA) equipped with a refractive index detector along with a Supelguard pre-column with dimensions of 5 cm × 4.6 mm (Supelco. Inc Bellefonte, PA, USA) and a Supelcogel TM C-610H column (30 cm × 7.8 mm). This method consisted in the homogenization of 1 mL of sample juice with phosphate buffer (5 mL) followed by filtration and injection. Sugars from Sigma (Poole.Dorset. UK) were used for calibration curves and quantification with a satisfactory linearity level (R^2^ ≥ 0.999). Analyses were run in triplicate and expressed as g L^−1^.

##### 2.3.2.7 Protein profile

Juice protein profile was assessed according to the modified method of Vincenzi et al. (2005). Thus, the juice proteins were precipitated using trichloroacetic acid at 4 °C. Then a volume of 20% trichloroacetic acid solution was added to 1 mL of each filtered juice sample. After 30 min, a centrifugation was applied at 14000 rpm for 15 min. The pellets obtained were washed with acetone solution and then suspended in ultrapure water. To quantify the protein content the standard Bradford test was used. In addition, standard reagent grade bovine serum albumin solutions (Equitech-Bio, Inc.) were prepared. The protein content of each sample was determined by fitting a least squares regression curve of the amount of standard protein concentration versus absorbance. Each sample was converted to grams per liter of juice.

##### 2.3.2.8 Organic acid profiles

The samples were kept at -20 °C until the time of analysis. The measurements concerned contents of citric, malic and quinic acids following the method previously described by Hernández et al. (2015) with few modifications. Indeed, samples of 0.5 g were mixed with of Milli-Q water (5 mL) and left for 30 min under ultra-sonication (UP 400St (400 W, 24 kHz), using Hielscher's digital ultrasonicator. The mixture was subjected to centrifugation for 20 min at 15,000 rpm (Sigma 3–18 K; Sigma, Osterode am Harz, Germany), and the supernatant was filtered through a 0.45 μm Millipore filter and used for analysis. All extractions were carried out in triplicate. The chromatographic analysis was carried out according to Hernández et al. (2015). Thus, 10 μL of aforementioned extract were injected into Supelcogel column (TM C-610H column 30 cm × 7.8 mm) of the same HPLC brand previously described coupled with diode-array detector (DAD) and equipped with an autosampler and a UV detector, set at 210 nm and a Supelguard pre-column (5 cm × 4.6 mm; Supelco, Bellefonte, PA). Phosphoric acid at a concentration of 0.1% and at a flow rate of 0.5 mL min ^−1^ was used as the elution buffer. Calibration curves displayed a very satisfactory linearity level (R^2^ ≥ 0.999) under a wavelength of 210 nm using. Concentrations were calculated as g kg^−1^ of dry weight.

##### 2.3.2.9 Fatty acids in seed oil

Pomegranate seeds oil was extracted using Soxhlet apparatus following NF NE ISO 659 reference method (AFNOR, 2009). Thus, twenty-five grams of each seeds powder genotypes were mixed with 250 mL of n-hexane (99%) as an oil extracting solvent using cellulose extraction thimbles (123 × 43 mm, Whatman International, Brentford, UK). The solvent was evaporated at 40 °C using a rotavapor. The resulting oil samples were stored in darkness at 4 °C until analysis to avoid their oxidation.

The methyl esters of the pomegranate seed oil were prepared in n-heptane (0.12 g/2 mL) with a cold KOH solution (2M) according to standard NF EN ISO 5509. The obtained fatty acid esters were analyzed using an Agilent Technologies 7890A gas chromatograph (GC) equipped with a flame ionization detector (T = 250 °C). The column used was a 60 m × 0.25 mm silica capillary column. The hydrogen inlet pressure of carrier gas was 178 kPa, with 1:70 ratio. The oven temperature program was as follows: 20 min at 210 °C, 210–245 °C at 6 °C/min then 10 min at 245 °C. A volume of 1 mL was injected with a split ratio of 1:50. The fatty acids methyl ester reference standard mixture (C4–C24, FAME Mix 37) was used for used for calibration and for the identification of the FAME by their retention times.

### 2.4 Statistical analysis

Prior to statistical analysis, data were standardized (μ = 0 and σ = 1) so they can have a comparable scale, using SPSS v22 software. Analysis of variance was performed to verify significant differences between the samples collected, except for fatty acids, as they only were determined once. The correlation heatmap was built using Pearson model by Originlab Pro v9. Principal component model was performed using the correlation coefficient and the Varimax rotation method with Kaiser normalization. In addition, a scatterplot was created according to the first two components of the principal component analysis (PCA) using SPSS v22 software. Two-dimensional clustered heatmap groups similar rows and similar columns together, with their similarity represented by a dendrogram. The data were clustered by Ward's method with the Euclidean distance. This method is of importance to achieve a better understanding of complex biological systems where one-way direction is assumed (Hssaini et al., 2021).

## 3 Results and discussion

### 3.1 Descriptive analysis

#### 3.1.1 Morphometric characterization

Variance analysis of fruit morphometric data are summarized in Table 2. Very large variability was observed within the genotypes regarding all examined traits, with the exception of seed weight. Thus, the fruit weight varied from 168.0 to 558.0 g, with an average of about 339.5 g. Fruit and calyx diameters varied from 67.2 to 99.4 mm and 15.4–29.4 mm, respectively. The total height of fruit with calyx and without calyx varied from 70.8 to 107.6 mm and from 55.2 to 86.8 mm, respectively. Similarly, the calyx height varied from 12.4 to 23.0 mm, while the number of carpels oscillated between 5.7 and 8.0, with an overall mean of 6.9. The bark weight and thickness oscillated from 35.0 to 200.0 g and from 1.2 to 3.4 mm, respectively. As for the weight of the seeds and the aril yield, they ranged from 27.5 to 80.0 g and from 35.0 to 92.9%, respectively. For the percentage in seeds weight, it was observed a variation from 8.2 to 21.7%. For aril weight, length and diameter, average values were in the following ranges of 0.15–0.52 g, 9.5–12.5 mm and 6.0–8.6 mm, respectively. Similarly, the seeds weight, length and diameter varied within the following ranges 0.03–0.11 g, 6.1–7.8 mm and 2.1–3.4 mm, respectively. According Khadivi et al. (2020), fruit size is an important criterion for consumers as well as for transportation. The scientific literature has shown a wide range of fruit weight variation among genotypes. In Italy, Turkey and Spain, similar works reported fruit weight ranges of 244–560 g, 200–610 g and 333–464 g, respectively (Durgaç et al., 2008; Hernández et al., 2014; Adiletta et al., 2018). Looking at the calyx, Khadivi et al. (2020), in Iran, indicated that the fruit calyx length ranged from 3.0 to 14.7 mm, while the calyx diameter oscillated between 4.8 and 23.0 mm. They also reported a range of 0.06–0.27 g for the weight of a single aril. In comparison to studies above cited, it is clearly noted that the pomological variations recorded on the 14 cultivars herein evaluated fall within the variation intervals for each single trait with evidently a remarkable phenotypic effect with regards to the environment effect as well.

**Table 2:** Descriptive analysis and analyze of variance of morphometric traits of the studied pomegranate accessions.

	Min	Max	Mean	Std. deviation	Mean square	ANOVA p-value
Fruit weight (g)	168.0	558.0	339.5	89.3	21951.3	<.0001
Fruit diameter (mm)	67.2	99.4	84.6	7.2	146.9	<.0001
Calyx diameter (mm)	15.4	29.4	19.9	2.7	17.8	<.0001
Fruit height without calyx (mm)	55.2	86.8	73.6	7.4	153.8	<.0001
Fruit height with calyx (mm)	70.8	107.6	88.5	8.1	177.5	<.0001
Calyx height (mm)	12.4	23.0	14.9	2.0	7.9	<.0001
Number of carpels	5.7	8.0	6.9	0.5	0.5	<.001
Bark weight with endocarp (g)	35.0	200.0	103.2	42.0	4347.7	<.0001
Bark thickness (mm)	1.2	3.4	2.1	0.5	0.5	<.005
Aril yield (%)	35.0	92.9	73.7	10.4	202.0	<.001
Juice yield (mL Fruit^−1^)	78.0	280.0	147.5	37.4	3647.7	<.0001
Total aril weight (g)	27.5	80.0	47.8	11.2	229.9	<.001
% of seed weight per aril	8.2	21.7	14.2	3.3	28.8	<.0001
Single aril weight (g)	0.15	0.52	0.36	0.08	0.02	<.0001
Aril length (mm)	9.5	12.5	10.9	0.7	1.6	<.0001
Aril diameter (mm)	6.0	8.6	7.2	0.6	1.1	<.0001
Seed weight (g)	0.03	0.11	0.04	0.01	0	0.067
Seed length (mm)	6.1	7.8	7.0	0.4	0.4	<.0001
Seed diameter (mm)	2.1	3.4	2.6	0.3	0.2	<.001

#### 3.1.2 Juice biochemical traits

Results regarding biochemical characterization are summarized in Table 3. All variables showed highly significant differences (p < 0.001) among studied samples. Hence, pomegranate juice seemed to be highly acidic, with pH values of 2.39–4.00 with an average of 3.22. Similar pH ranges were previously reported in the scientific literature. Indeed, the study carried out in Spain by Fernandes et al. (2017) have reported very similar results, where pH values ranged from 2.56 (cv. Wonderful) to 4.31 (cv. Valenciana). Other similar works reported pH ranges of 2.81–3.90, 3.49–5.14, 2.93–3.59, 3.16–4.09, 3.94–4.07, 2.9–4.0 and 2.98–3.68 (Ozgen et al., 2008; Tehranifar et al., 2010; Gadže et al., 2012; Legua et al., 2012; Ferrara et al., 2014; Melgarejo-Sánchez et al., 2015; Raduníc et al., 2015). However, it is important to emphasize that several factors related to genotype, climate and agricultural practices may explain differences in terms of juice pH. Among the studied cultivars, ‘Zheri Precoce’, ‘Djeibi’ and ‘Chelfi’ showed the most acidic pH, of 2.39, 2.76 and 2.64, respectively.

**Table 3:** Descriptive analysis of biochemical and lipo-chemical traits of the pomegranate accessions studied.

	Min	Max	Mean	Std. Deviation	Mean Square	ANOVA p-value
pH	2.39	4.00	3.22	0.44	0.59	<.0001
Total soluble solids (°Brix)	13.00	17.00	15.19	1.05	3.41	<.0001
Titrable acidity (g CAE L^−1^)	1.80	25.50	7.00	0.63	1.21	<.0001
Maturity index	0.54	8.11	3.81	0.22	14.78	<.0001
Juice dry matter (g L^−1^)	91.60	155.40	129.00	14.20	6.04	<.0001
Hydrolysable tannins (mg ​TAE L^−1^)	2.02	7.52	3.85	1.53	6.62	<.0001
Condensed tannins (mg ​TAE L^−1^)	2.13	8.13	5.12	1.59	7.77	<.0001
Total anthocyanins (mg CGE ​L^−1^)	49.00	280.00	108.47	50.12	6371.71	<.0001
Polyphenols (g GAE L^−1^)	1.49	9.67	5.45	2.35	17.38	<.0001
Flavonoids (g QE L^−1^)	0.13	0.47	0.28	0.08	0.02	<.0001
Proteins (g L^−1^)	1.35	8.85	4.20	2.59	21.01	<.0001
Sugars						
Glucose (g L^−1^)	29.43	96.96	70.77	24.21	1841.94	<.0001
Fructose (g L^−1^)	33.38	102.75	75.86	24.29	1854.43	<.0001

Organic acids						
Citric acid (g Kg^−1^)	0.59	11.60	2.67	2.49	19.25	<.0001
Malic acid (g Kg^−1^)	1.45	7.54	4.59	1.92	11.31	<.0001
Quinic acid (g Kg^−1^)	2.33	16.65	9.65	4.34	59.04	<.0001
Fatty acids (%)						
Capric acid	0.34	0.35	0.35	0.01	0	NA
Caproic acid	0.03	0.09	0.06	0.01	0	NA
Lauric acid	0.40	0.30	0.35	0.01	0	NA
Palmitic acid	1.77	7.64	4.18	2.19	4.80	NA
Palmitoleic acid	0.21	2.59	1.03	0.90	0.80	NA
Margaric acid	0.006	0.008	0.007	0	0	NA
Stearic acid	1.02	4.87	2.51	1.43	2.04	NA
Oleic acid	0.01	8.67	4.39	3.14	9.89	NA
Vaccenic acid	0.005	0.006	0.005	0	0	NA
Linoleic acid	0	4.49	2.70	1.39	1.92	NA
Alpha-linolenic acid	0.27	1.40	0.80	0.38	0.14	NA
Gadoleic acid	0.01	0.96	0.33	0.31	0.09	NA
Behenic acid	1.23	2.57	1.92	0.63	0.40	NA
Erucic acid	0.56	11.06	5.51	4.47	20.01	NA
Lignoceric acid	0.01	0.21	0.13	0.06	0	NA

CAE: citric acid equivalent; TAE: tannic acid equivalent; CGE: cyanidin-3-glucoside equivalent; GAE: gallic acid equivalent; QE: quercetin equivalent; NA: not applied (means that ANOVA test was not applied to these variables).

The total soluble solids content (TSS) showed a significant variability with an interval of variation of 13.00–17.00 °Bx. The highest TSS contents were recorded in the cultivars ‘Grenade Jaune’, ‘Chelfi’ and ‘Mollar Osin Hueso’, of which the average levels were 17.00, 16.20 and 16.06, respectively. The lowest levels were observed in the genotypes ‘Zheri Precoce’ (13.00 °Bx) and ‘Sefri’ (14.00 °Bx). The results are comparable to those found by Tehranifar et al. (2010) in cultivated pomegranates (15.17–22.03 °Bx). Hasnaoui et al. (2011) also reported similar TSS values in wild and cultivated pomegranate fruits, with respective ranges of 17.57–19.99 °Bx and from 13.13 to 16.55 °Bx. The herein TSS range of variation is comparable to that described in previous studies held in Spain, where the climate is quite similar to that of northern Morocco. Thus, TSS was reported between 14.31 and 15.81 °Bx (Melgarejo et al., 2011), 14.79–15.81 °Bx (Legua et al., 2012), 11.94–14.84 ºBx (Melgarejo et al., 2012), 13.73–17.60 °Bx (Mena et al., 2011) and 12.36–16.32 °Bx (Martínez et al., 2012). Note that the high content of TSS is a decidedly desirable trait for fresh consumption as well as for the food industry, especially in pomegranate juice processing as it combines sweetness and flavor (Khadivi et al., 2020).

Titratable acidity (TA) also revealed significant variability within genotypes, ranging from 1.80 to 25.50 g CAE L^−1^ with a mean value of 7.00 g CAE L^−1^. The TA was found to be higher in the local genotypes ‘Sefri2’, ‘Djeibi’ and ‘Bzou’, with average values of 25.50, 15.00 and 12.50 g CAE L^−1^, respectively. However, TA was substantially low in the exotic genotypes ‘Gordo de Jativa’, ‘Dwarf Semi Evergreen’ and ‘Zheri Precoce’, with respective averages of 1.80, 2.40 and 2.50 g CAE L^−1^. The levels herein reported for TA were generally similar to those reported by Nuncio-Jáuregui et al. (2014); Calín-Sánchez et al. (2011) and Dandachi et al. (2017). The fact that the results obtained seem to have larger intervals of variation than those found in previous studies can be attributed to genetic differences, in addition to environmental factors and agricultural practices (Adiba et al., 2021). Pomegranate maturity index (MI) of juice is an important indicator in assessing its quality and internal flavor. Thus, the higher the index, the sweeter the fruit and the lower the index, the more acidic the taste (Colaric et al., 2005). The MI values varied largely among genotypes (0.54–8.11), showing a great phenotypic variability in terms of fruit sweetness. The cultivars ‘Gordo de Jativa’, ‘Dwarf semi-Evergreen’, ‘Chioukhi’ and ‘Grenade Jaune’ were the sweetest with mean MI of 8.11, 6.19, 5.94 and 4.92, respectively. However, ‘Sefri2’ (0.54), ‘Djeibi’ (1.02) and ‘Bzou’ (1.21) were the most acidic (Table 3). Our results are in agreement with those of Mena et al. (2011) who reported MI values ranged from 4.31 to 38.62. In addition, Fernandes et al. (2017) indicated that when MI is between the values of 11 and 16, pomegranates are considered to be quite tasty, which is the case of all the local genotypes herein studied. The variation in this index refers to the balance between the increase in sugar concentration and the decrease in titratable acidity during the ripening processes (Melgarejo et al., 2012).

A very important range of diversity was observed for the two characters "hydrolysable tannins" and "condensed tannins" whose extreme values recorded respectively 2.02–7.52 mg TAE L^−1^ and 2.13–8.13 mg TAE L^−1^. Similarly, the genotypes studied were shown to have a very significant variability for the concentration of polyphenol ranging from 1.49 to 9.67 g GAE L^−1^ and for flavonoids ranging from 0.13 to 0.47 g QE L^−1^. The highest polyphenol concentrations were recorded by the genotypes ‘Grenade Jaune’, ‘Chioukhi’ and ‘Gordo de Jativa’, where the average values were respectively of 9.67, 8.80 and 8.29 g GAE L^−1^. Further, the lowest levels were recorded by the genotypes ‘Dwarf Semi Evergreen’ and ‘Zheri Precoce’ which were marked by the averages 1.49 and 1.56 g GAE L^−1^. The results herein found are comparable to those of Fernandes et al. (2017) regarding total flavonoids content, ranging from 0.21 to 1.89 g QE L^−1^ over nine Spanish pomegranate varieties. Similar intervals were also reported by Thakur et al. (2010), Sharma and Thakur (2016) and Sharma and Thakur (2018). Total anthocyanins ranged from 49.00 to 280.00 mg CGE L^−1^ with an average of 108.47 mg CGE L^−1^. Local genotypes, ‘Djeibi’, ‘Grenade Jaune’ and ‘Sefri2’ exhibited the highest anthocyanins contents of 280.00, 161.58 and 155.16 mg CGE L^−1^, respectively (Table 3). Singh and Sethi (2003) recorded a large variation in anthocyanins on seven pomegranate cultivars, ranging from 10.30 to 17.85 mg 100 g^−1^ of fruit.

Pomegranates are generally appreciated for their taste and their sweetness which can be influenced by their content in sugars, mainly glucose and fructose. In fact, the glucose and fructose concentrations varied successively from 29.43 to 96.96 g L^−1^ and from 33.38 to 102.75 g L^−1^, with an overall average of 70.77 and 75.86 g L^−1^. The highest mean values of glucose and fructose respectively were recorded by two local genotypes, ‘Djeibi’ (96.96; 102.75 g L^−1^) and ‘Bzou’ (91.44; 97.37 g L^−1^) as well as by the exotic variety ‘Ruby’ (91.79; 96.23 g L^−1^). The genotype ‘Wonderful’ from the United States was characterized by the lowest concentration of glucose (29.43 g L^−1^) and fructose (33.38 g L^−1^). In general, the glucose and fructose levels in the pomegranate collection are comparable to those of previous works (Melgarejo et al., 2000; Al-Maiman and Ahmad, 2002; Ozgen et al., 2008; Cam et al., 2009; Shwartz et al., 2009; Bar-Yaakov et al., 2019). Furthermore, the studied genotypes were marked by a wide range in protein content, ranging from 1.35 to 8.85 g L^−1^, with an overall average of 4.20 g L^−1^. The highest values were recorded by the local genotypes ‘Chelfi’, ‘Sefri2’ and ‘Zheri Precoce’, with mean concentrations of 8.85, 7.38 and 6.12 g L^−1^, respectively (Table 3).

#### 3.1.3 Organic acid profiles

Ctric acid concentration was found between 0.59 and 11.60 g kg^−1^ with an overall mean of 2.67 g kg^−1^ (Table 3). The genotypes ‘Negro Monstrioso’ and ‘Sefri2’ showed the highest values, of 11.6 and 4.20 g kg^−1^, respectively. The lowest concentrations were recorded by the genotypes ‘Sefri’ (0.59 g kg^−1^), ‘Wonderful’ (0.82 g kg^−1^) and ‘Chelfi’ (0.99 g kg^−1^). These results are similar to those reported by Fernandes et al. (2017). The malic acid also showed a very remarkable fluctuation between genotypes ranging from 1.45 to 7.54 g kg^−1^, with an average of around 4.60 g kg^−1^. The genotypes marked by high concentrations of malic acid were ‘Ruby’ (7.54 g kg^−1^), ‘Djeibi’ (6.83 g kg^−1^) and ‘Dwarf Semi Evergreen’ (6.72 g kg^−1^). In addition, our data showed that the two local genotypes ‘Sefri2’ and ‘Djeibi’ were identified by their high concentrations in quinic acid, with respective mean values of 16.65 and 14.10 g kg^−1^. The variation range for this acid was 2.33–16.65 g kg^−1^, with an average of 9.65 (g Kg^−1^). The results obtained seem to present larger variation intervals than those described in prior studies (Melgarejo et al., 2000; Cam et al., 2009; Shwartz et al., 2009). Large differences were found for citric and malic acid contents compared to those reported by Melgarejo et al. (2000). Previous studies have noted that ascorbic, quinic, succinic, acetic, lactic and fumaric acids have been detected in aril juice in minor amounts or in trace amounts (Poyrazglu et al., 2002). Rajasekar et al. (2012) revealed that citric acid and malic acid have been identified as the predominant pomegranate juice organic acids. Usually, citric acid is the main acid known in pomegranate genotypes (Legua et al., 2016), providing the sour taste (Bar-Yaakov et al., 2019). Moreover, Melgarejo et al. (2011) indicated that both citric, oxalic, acetic, fumaric and tartaric acids are among the main organic acids found in pomegranate juice.

#### 3.1.4 *Fatty acid* profiles

Pomegranate seeds were reported to hold an important amount of lipids, of which the concentration varies between 140 to 270 g kg^−1^ (Jing et al., 2012; Amri et al., 2017). The quality parameters for today's consumer are the fat content and fatty acid composition and in particular, the ratio of saturated and unsaturated fatty acids and the balance between n-6 and n-3 long chain fatty acids. The composition of fat in fruits and their seeds has recently gained a great interest, especially due to the health promoting effects of their essential fatty acids including linoleic, linolenic and arachidonic acids with more emphasis on polyunsaturated fatty acids. This is mainly due to their role in preventing cardiovascular disease and other heart problems, as polyunsaturated fatty acids reduce HDL cholesterol levels (Stowe, 2011; Aviram and Rosenblat, 2012).

Analysis of the results revealed large differences in fatty acid concentrations between the studied seed oil samples. The highest averages were recorded for erucic acid (5.51%); palmitoleic acid (1.03%); oleic acid (4.39%); palmitic acid (4.18%), linolenic acid (2.7%) and gadoleic acid (0.33%). The genotypes ‘Mollar Osin Hueso’ and ‘Djeibi’ were characterized by the highest concentrations of erucic acid, the average concentrations of which were 10.83 and 11.06%, respectively. On the other hand, ‘Negro Monstrioso’ and ‘Chelfi’ were marked by the lowest concentrations, less than 0.90%. Likewise, for oleic acid, the Spanish genotypes ‘Gordo de Jativa’ and ‘Negro Monstrioso’ were characterized by a high concentration of oleic acid, with respective mean values of 8.67 and 8.44%. As for palmitoleic acid, it has been found in high concentrations in the two exotic genotypes ‘Ruby’ (2.59%) and ‘Negro Monstrioso’ (1.55%). On the other hand, the local genotypes displayed generally significant concentrations in some fatty acids, such palmitic acid fairly present in ‘Sefri2’ (3.82%), behenic acid in ‘Chioukhi’ (1.56%) and erucic acids in ‘Grenade Jaune’ (1.41%).

### 3.2 Principal component analysis

The principal component analysis (PCA) was used to identify the most significant descriptors of the data set. Only a variance greater than ǀ0.5ǀ was considered significant for each factor. Thus, total variance of 96.97% was explained by the first 11 principal components (PCs). The first four PCs included 37 variables representing more than 74% of all variables, and explained 59.79% of the total variance (Table 4), which means that these attributes showed the greatest variation between genotypes and had the greater impact on their discrimination. The first component accounted for 22.47% of the total variance, which is strongly influenced by 17 parameters of which 13 are morphometric and 4 biochemical parameters. Thus, for morphometric parameters, there are: fruit weight (r = 0.86 ∗∗), fruit diameter (r = 0.94 ∗∗), Calyx diameter (r = 0.63∗∗), fruit height without calyx (r = 0.97∗∗), bark weight with endocarp (r = 0.64∗∗), seed yield (r = 0.5∗∗), juice yield (r = 0.93∗∗), % of seed weight per aril (r = -0.75∗∗), single aril weight (r = 0.59), seed weight (r = 0.68∗∗), seed length (r = 0.6∗∗) and seed diameter (r = 0.61∗∗). For the biochemical parameters of the first component we found the contents of anthocyanins (r = 0.54∗∗) along with margaric (r = -0.55), vaccenic (r = -0.72∗∗) and alpha-linolenic (r = -0.65∗∗) acids. Taking into account the number of accessions evaluated, the above-described correlations are statistically satisfactory, and indicating that PC1 classify pomegranate genotypes based on almost all herein studied morphometric traits (13 traits) along with four biochemical attributes. The second function represents 15.86% of the total variance and is mainly explained by the following biochemical parameters: hydrolysable tannins (r = 0.65∗∗), citric (r = 0.82∗∗), palmitic acid (r = 0.89∗∗), palmitoleic acid (0.74∗∗), stearic acid (r = 0.89∗∗), oleic acid (r = 0.86∗∗), linoleic acid (r = 0.76∗∗) and behenic acid (r = -0.63∗∗). The third function represents 11.58% of the total variation which is related primarily to the aril length (r = -0.64), aril diameter (r = -0.72), total soluble solids (r = 0.57), titratable acidity (r = 0.59), maturity index (r = -0.57), dry matter (r = -0.64) and lignoceric acid (r = -0.56) (Table 4). Finally, the last component represents 9.88% of the total variation, which is associated to number of carpels (r = -0.51∗∗), malic acid (r = -0.76), quinic acid (r = -056), glucose and fructose (−0.72). In general, these results were in accordance with those of previous reports on different accessions from those herein studied, thereby indicating that the highlighted relationships are often encountered in the pomegranate species. These references highlight the importance of morphometric characterization as a key factor in the discrimination and evaluation of pomegranate genotypes. Khadivi et al. (2020) also reported that the morphometric descriptors are to be strongly considered in assessing the diversity of pomegranate accessions. In addition, they reported that the selection of highly discriminating variables is of great importance in optimizing resources for the morphometric characterization of edible pomegranate.

**Table 4:** Correlation coefficients between the main PCA components and the observed variables.

Variables	Principal components (PC)								
	PC 1	PC 2	PC 3	PC 4	PC 5	PC 6	PC 7	PC 8	PC 9
Fruit weight	**0.86**	-0.03	0.33	0.08	0.19	0.14	0.24	0	0.01
Fruit diameter	**0.94**	0.04	0.15	0.02	0.11	0.01	0.13	0.12	0.14
Calyx diameter	**0.63**	-0.28	-0.18	-0.16	-0.37	0.25	0.23	0.43	-0.04
Fruit height	**0.97**	0.08	0.18	-0.02	0.05	0.10	-0.01	0	0.07
Height of the fruit without calyx	**0.97**	0.01	0.16	0.02	-0.08	0.14	-0.01	0.03	0.02
Height of the chalice	0.45	-0.27	0.01	0.21	-0.66	0.26	-0.05	0.15	-0.24
Number of carpels	0.28	-0.35	-0.29	**-0.51**	0.25	-0.30	0.15	0.36	0.23
Weight of bark and endocarp	**0.64**	0.16	0.11	0.25	0.11	0.21	0.26	0.53	-0.07
Bark thickness	0.25	0.27	0.37	0.13	-0.61	-0.15	-0.18	0.26	0.24
Aril yield	**0.50**	-0.17	0.37	-0.29	-0.12	-0.29	0.09	-0.45	0.20
Juice yield	**0.93**	0	0.24	0.09	0.05	-0.05	0.16	-0.07	-0.02
Aril weight	0.42	-0.25	0.37	0.49	0.45	-0.07	-0.17	-0.02	-0.07
% of seed weight per aril	-**0.75**	-0.13	-0.06	0.4	0.38	-0.14	-0.05	-0.17	-0.2
Single aril weight	**0.59**	-0.29	-0.01	0.42	0.08	0.13	-0.05	0.11	-0.18
Aril length	0.36	-0.10	**-0.64**	0.23	0.26	-0.14	0.49	-0.02	0.11
Aril diameter	0.46	-0.18	**-0.72**	0.26	-0.07	-0.15	0.19	0.16	0.14
Seed weight	**0.68**	0.16	0.05	0.37	-0.17	0.50	-0.21	0.08	-0.04
Seed length	**0.6**	-0.07	-0.23	0.28	0.36	-0.04	0.31	-0.15	0.38
Seed diameter	**0.61**	0.17	-0.36	0.41	-0.31	0.05	-0.29	-0.10	0.03
pH	0.06	0.02	0.02	0.43	0.45	0.55	-0.08	0.14	-0.27
Total soluble solids	-0.12	-0.32	**0.57**	0.15	0.06	0.21	-0.5	0.04	0.45
Titratable acidity	0.26	0.37	**0.59**	-0.27	0.09	-0.48	0	-0.05	-0.29
Maturity index	-0.33	-0.39	**-0.57**	0.05	0.10	0.52	0.26	-0.06	0.19
Dry matter	-0.03	0.29	**-0.64**	0.2	-0.29	-0.30	0.12	-0.12	-0.46
Hydrolysable tannins	-0.11	**0.65**	0.43	0.17	-0.5	0.19	-0.02	-0.15	0.07
Condensed tannins	-0.14	0.17	0.41	-0.32	-0.49	-0.33	0.38	0.13	0.10
Total anthocyanins	**0.54**	0.08	0.39	0.1	0.48	-0.3	-0.06	0.05	-0.12
Total polyphenols	-0.26	-0.10	0.37	0.29	0.55	0.54	0.15	0.17	0.02
Total flavonoids	-0.1	-0.24	0.06	0.03	0.84	-0.25	0.03	-0.05	0
Proteins	-0.3	0.18	0.29	0.15	0.02	-0.08	-0.47	0.38	-0.04
Glucose	0.29	0.29	-0.12	**-0.72**	0.38	0.25	-0.2	-0.01	-0.03
Fructose	0.36	0.25	-0.11	**-0.72**	0.36	0.22	-0.21	-0.05	0
Citric acid	-0.11	**0.82**	0.14	0.15	-0.1	0.19	0.10	-0.32	0.21
Malic acid	0.28	0.28	-0.19	**-0.76**	0.24	0.23	-0.33	0.01	-0.05
Quinic acid	0.44	0.46	0.29	**-0.56**	0.14	0.28	-0.24	-0.18	0.05
Caproic acid	0.07	-0.28	-0.24	0.39	0.05	-0.47	-0.23	-0.13	0.47
Capric acid	0.18	-0.15	-0.05	0.32	0.21	-0.47	-0.46	-0.06	0.07
Lauric acid	0.39	0.34	0.36	-0.25	0.10	-0.22	0.50	-0.08	-0.02
Palmitic acid	-0.01	**0.89**	-0.36	0.21	0.08	0.08	0	0.07	0.08
Palmitoleic acid	-0.07	**0.74**	-0.24	0.12	0	0.36	-0.2	0.04	0.17
Margaric acid	**-0.55**	-0.04	0.22	-0.14	0.29	0.16	0.05	0.59	0.18
Stearic acid	-0.04	**0.89**	-0.35	0.22	0.06	0.1	0.01	0.06	0.09
Oleic acid	-0.04	**0.86**	-0.44	0.12	0.15	-0.01	0.06	0.09	0.08
Vaccenic acid	**-0.72**	0.09	0.11	-0.13	-0.01	-0.10	0.02	0.59	-0.06
Linoleic acid	0.13	**0.76**	-0.43	-0.26	0.2	-0.11	0.16	0.12	-0.23
Alpha-linolenic acid	**-0.65**	0.09	0.37	0.23	0.28	0.3	0.32	-0.08	0.16
Gadoleic acid	-0.11	0.90	-0.23	0.09	0.16	-0.11	0.02	-0.2	0.18
Behenic acid	-0.12	**-0.63**	0.06	-0.10	-0.16	0.53	0.24	-0.42	-0.08
Erucic acid	0.49	0.01	-0.30	0.22	0.19	-0.49	-0.52	0.10	-0.21
Lignoceric acid	0.05	-0.29	**-0.56**	-0.35	-0.22	-0.06	-0.25	0.27	0.38
% of variance	22.47	15.86	11.58	9.88	8.85	8.02	5.89	4.94	3.50
Cumulative variance %	22.47	33.33	49.91	59.79	68.64	76.66	82.55	87.49	90.99

Significant correlation coefficients were marked in bold.

### 3.3 Scatter plot

**Figure 1:**
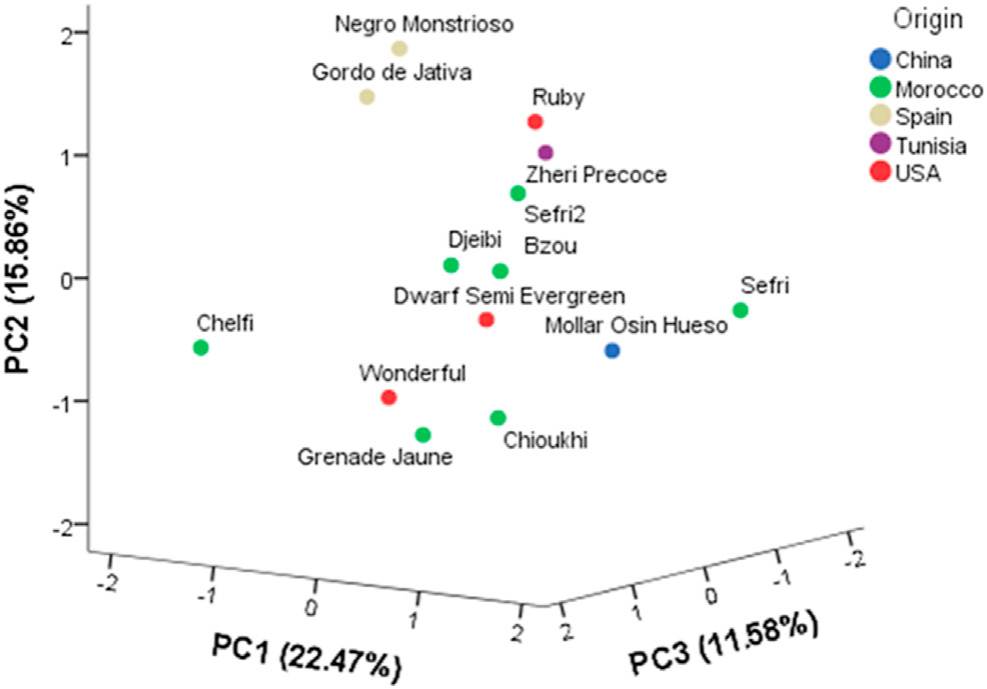
Scatterplot of the first three principal components for the pomegranate collection studied, based on all descriptors.

The scatter plot was prepared as a function of the first three principal components, PC1, PC2 and PC3 whose variance was respectively 21.76, 16.12 and 11.96% and which distinguish genotypes according to their phenotypic characteristics (Figure 1). The strong phenotypic variation revealed within the pomegranate genetic material involved in this study can be attributed to the genetic factor as long as the accessions studied belong to an ex-situ collection conducted under the same edaphoclimatic conditions. Thus, genotypes are generally separated into three groups. The first group brings together five Moroccan genotypes ‘Sefri’; ‘Bzou’; ‘Grenade Jaune’; ‘Chioukhi’ and ‘Djeibi’ indicating the authenticity of the local genotypes except the genotypes ‘Chelfi’ and ‘Sefri 2’, which were clustered in different subcluster and largely distinguished from the group. The second group was accounted for four genotypes (‘Dwarf Semi Evergreen’, ‘Wonderful’, ‘Mollar Osin Hueso’ and ‘Chelfi’). Two of which are originated in USA, one in Morocco and the other in China. The third cluster includes four genotypes, two of which are from Spain (‘Ruby’ and ‘Gordo de Jativa’), one from Tunisia (‘Zheri Precoce’) and the last one from Spain (‘Negro Monstrioso’). This study suggested a great diversity in morphological and physicochemical characters and could be used as an effective first approach for the discrimination of genotypes of pomegranate.

### 3.4 Correlation between variables

**Figure 2:**
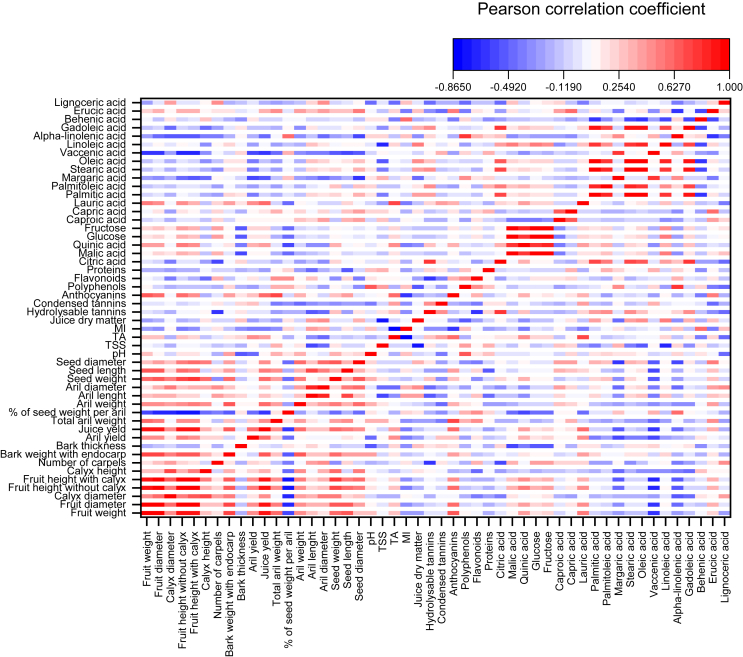
Bivariate split heatmap correlations among the morphometric and lipo-biochemical characters in the studied 14 genotypes of pomegranate.

In order to identify the relationships between the morphometric, biochemical and lipo-chemical traits studied, all mean values were involved in the bivariate split heatmap correlation using the Pearson model. The most potential and significant correlations are summarized in Figure 2. Indeed, positive correlations being statistically highly significant with a high weight were marked in red, while the highest negative correlations are those marked in blue.

Results show very strong positive and significant correlations related to fruit weight with diameter, height, fruit height without calyx, bark weight, juice yield, seed weight and seed length. These results are similar to those described in prior studies (Khadivi-Khub et al., 2015; Khadivi et al., 2018). Likewise, other correlations between height of the fruit without calyx with weight, diameter, calyx diameter, and fruit height were observed. In addition, strong positive correlations related the juice yield with weight, diameter, fruit height and with height of the fruit without calyx. Previous reports have shown similar results (Khadivi et al., 2020). This indicates that the genotypes marked by a high juice yield are those characterized by a large total diameter and a large fruit weight. Likewise, the height of the fruit without calyx, bark weight, and seed length are strongly related with weight, with diameter, and with fruit height. Similar results in previous works have been shown (Legua et al., 2012; Khadivi et al., 2018). On the other hand, negative and significant correlations were also recorded between percentage in seed weight per aril and the following parameters: fruit weight, fruit diameter, calyx diameter, fruit height, fruit height without calyx and juice yield. Other negative and highly significant correlations are also recorded between vaccinic acid with fruit weight, diameter, height, height of the fruit without calyx, seed yield, juice yield and seed length. For biochemical properties, relationships between biochemical attributes were investigated to assess possible correlations within this group of data. This analysis has particularly focused on the relationships between fatty acids and other parameters of agronomic interest. In addition, other correlation considered important between palmitoleic acid and stearic acid, as well as between oleic acid and gadoleic acid. Important correlations link stearic acid to oleic, linoleic, gadoleic and citric acids. Positive correlations have been recorded between oleic acid and linoleic acid, as well as between gadoleic acid and citric acid. Malic acid concentration was also correlated with quinic acid, glucose and fructose contents. Fructose content was very strongly correlated with glucose content. Negative and highly significant correlations are also recorded between behenic acid and palmitic, stearic, oleic, linoleic and gadoleic acids. Margaric and alpha-linolenic acids levels were negatively linked to seed diameter. The observed correlation can provide information on potentially important descriptors for the assessment of genotypes. Significant and strongly relationships can be further investigated for a preliminary and rapid prediction of other traits and thus could be recommended in future screening works particularly with a larger sample length.

### 3.5 Two dimensional clustered heatmap

**Figure 3:**
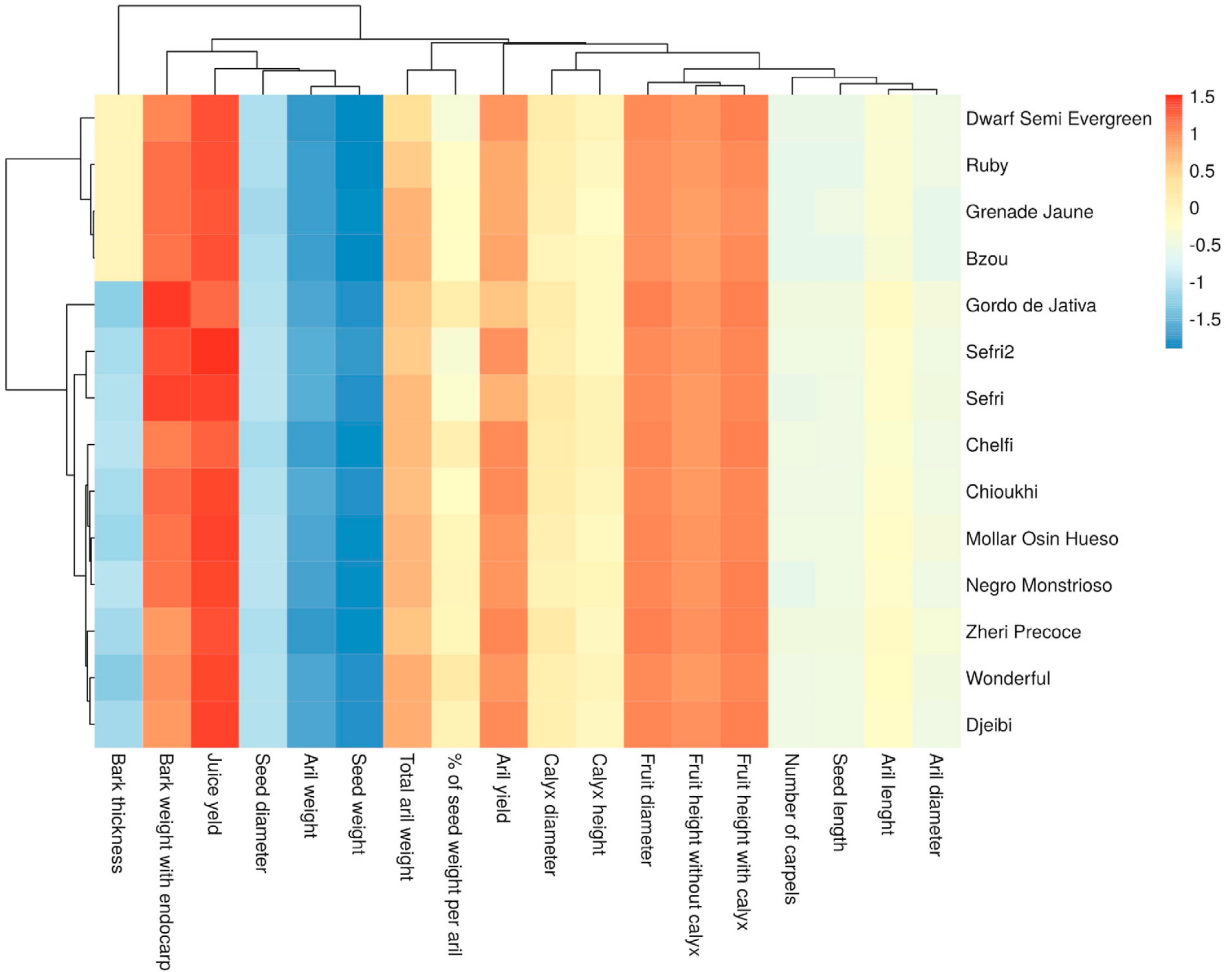
Cluster analysis of the studied pomegranate genotypes based on morphometric traits. Each small square reflects the phenotypic characteristics of pomegranate genotypes. The color represents the normalized value with red representing the larger value and blue representing the lower value. Each row represents the normalized content of different phenotypic characteristics from one genotype. Each column represents the difference in the normalized results of different genotypes in a single specific phenotype.

**Figure 4:**
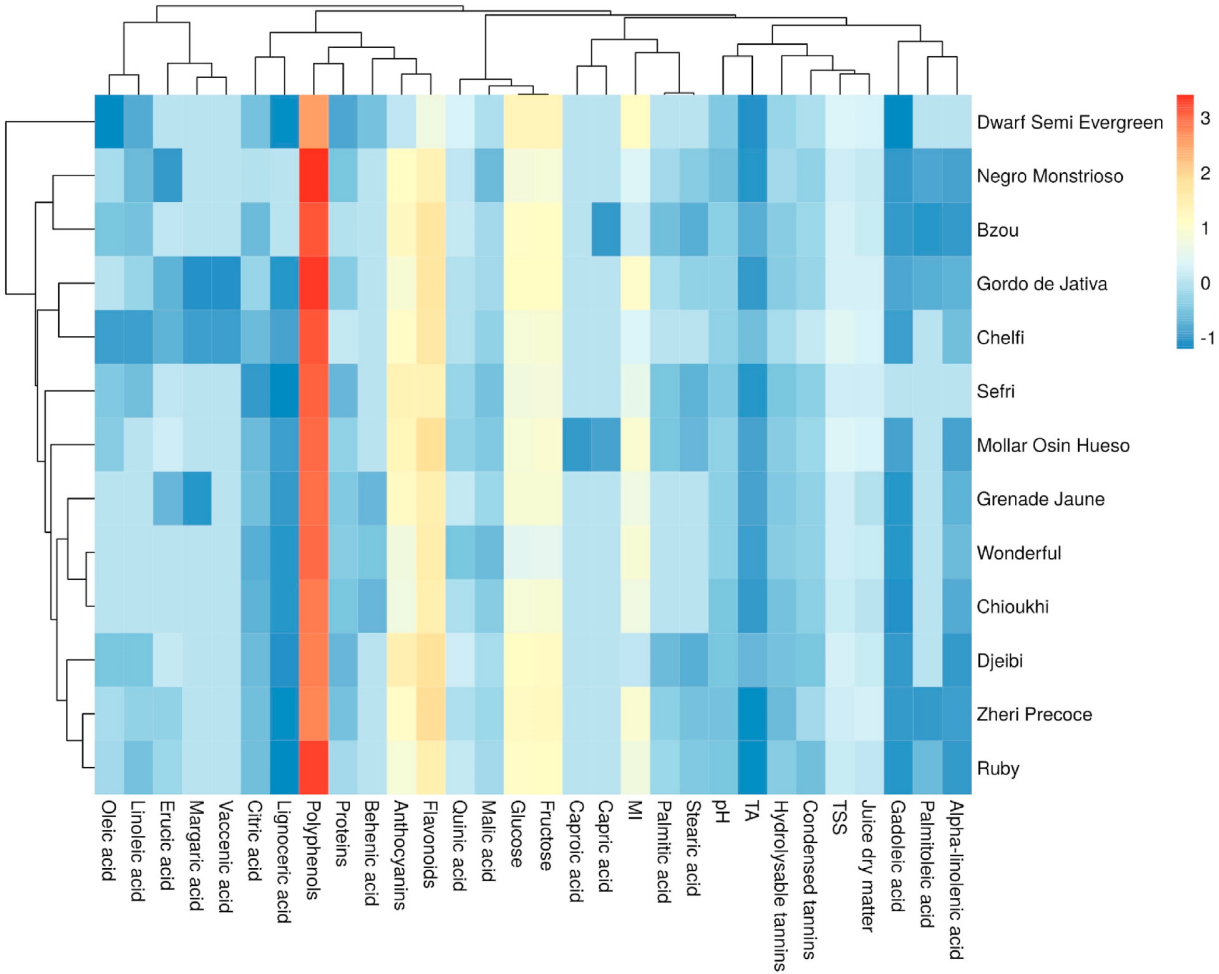
Cluster analysis of the studied pomegranate genotypes based on lipo-biochemical traits. Each small square reflects the phenotypic characteristics of pomegranate genotypes. The color represents the normalized value. with red representing the larger value and blue representing the lower value. Each row represents the normalized content of different phenotypic characteristics from one genotype. Each column represents the difference in the normalized results of different genotypes in a single specific phenotype.

In order to obtain a simplified classification of the studied genotypes using all morphometric parameters (Figure 3) and lipo-biochemical traits (Figure 4), a two-dimensional heatmap clustering was generated; one is sample-oriented whereas the other is variable-oriented. Each small square represents the phenotypic or lipo-biochemical characteristics of pomegranate tree genotypes. The figures display a colored data matrix, which gives an overview of the numeric differences between studied samples. A weak effect on the dataset is displayed in low color intensity, while a stronger one is shown with high color intensity. Color materializes normalized value of the herein investigated traits, where the red color refers to high values, while the blue one describes lower values. Besides, for both colors, the intensity represents the strength of the effect. Each square indicates the normalized content of a wide range of traits from one cultivar. The morphometric traits based-heatmap, showed in Figure 3, highlighted that fruit diameter and juice yield had higher scores in the dataset, which means that they have a higher effect on genotypes distribution. However, seed weight, aril weight, seed diameter and bark thickness had a low impact on the dataset. The obtained dendrogram clearly differentiated five main clusters, where the exotic variety ‘Gordo de Jativa’ alone constitutes a separate group, which is particularly distinguished by the lowest fruit weight and juice yield. On the other hand, the heatmap based on lipo-biochemical data (Figure 4) showed seven distinct clusters, where contents in polyphenols, flavonoids, anthocyanins and tannins (hydrolysable and condensed) had the highest impact on discrimination between the studied cultivars. Among the obtained clusters, ‘Dwarf Semi Evergreen’ was singled out as a separate group, having shown the lowest contents in polyphenols and flavonoids. Comparable results were reported by Loukhmas et al. (2021) in screening study on ten pomegranate cultivars of widely known commercial interest in Morocco. Finally, both PCA and heatmap analysis helped to better understand the relationship between the variables and resulted clusters.

## 4 Conclusion

This study showed a great variability within the studied pomegranate accessions, particularly among local genotypes, indicating that Morocco is an important hotspot of pomegranate germplasm diversity. Morphometric and biochemical screening of the pomegranate collection evaluated in this study revealed a wide range of variation for all parameters. The results also displayed interesting correlations linking some parameters of interest to others, making thus future screening studies of this collection more efficient. The most discriminating variables in genotypes classification were fruit weight and its dimensions, as well as juice content and some organic acids, mainly citric, palmitic, stearic and oleic acids. This multivariate screening made it possible to identify similarities and dissimilarities between some exotic varieties and local cultivars. The obtained database showed that local cultivars demonstrated a great potential and might be preconized as parents in breeding programs as well as in promising valorization pathways owing to their fruit quality. However, one of the limitations of this work is that a larger sample length is required and further validation must be performed using samples from other varieties and origins and through conducting other assays in contrasted locations, so the stability of the performances herein highlighted could be verified. Nevertheless, this study provides extensive data of great interest regarding lipo-biochemical attributes of pomegranate seeds that can be exploited for nutritional, pharmaceutical and cosmetic purposes.

## Declarations

### Author contribution statement

Assia Ejjilani: Performed the experiments; Analyzed and interpreted the data; Wrote the paper.

Karim Houmanat; Lahcen Hssaini: Analyzed and interpreted the data; Wrote the paper.

Hafida Hanine: Conceived and designed the experiments; Contributed reagents, materials, analysis tools or data; Wrote the paper.

Kaoutar Elfazazi; Francisca Hernandez; Ilham Hmid: Contributed reagents, materials, analysis tools or data.

Rachid Razouk: Conceived and designed the experiments; Performed the experiments; Analyzed and interpreted the data; Wrote the paper.

### Funding statement

This research did not receive any specific grant from funding agencies in the public, commercial, or not-for-profit sectors.

### Data availability statement

Data will be made available on request.

### Declaration of interests statement

The authors declare no conflict of interest.

### Additional information

No additional information is available for this paper.
